# Particle Exposure Hazards of Visiting Outdoor Smoking Areas for Patients with Asthma or COPD Even in EU Countries with Comprehensive Smokefree Laws

**DOI:** 10.3390/ijerph20115978

**Published:** 2023-05-28

**Authors:** Sheila Keogan, Tamara Alonso, Salome Sunday, Joan Hanafin, Olena Tigova, Esteve Fernandez, Maria Jose Lopez, Silvano Gallus, Sean Semple, Anna Tzortzi, Roberto Boffi, Giuseppe Gorini, Angel Lopez-Nicolas, D. K. Arvind, Cornel Radu-Loghin, Joan B. Soriano, Luke Clancy

**Affiliations:** 1 TobaccoFree Research Institute Ireland (TFRI), D02 HW71 Dublin, Ireland; 2 Hospital Universitario de la Princesa, Universidad Autónoma de Madrid, 28049 Madrid, Spain; 3 CIBER Respiratory Diseases (CIBERES), 28029 Madrid, Spain; 4 Tobacco Control Unit, Catalan Institute of Oncology (ICO), 08908 Barcelona, Spain; 5 Tobacco Control Research Group, Bellvitge Biomedical Research Institute (IDIBELL), 08908 Barcelona, Spain; 6 School of Medicine and Health Sciences, Bellvitge Campus, Universitat de Barcelona, 08007 Barcelona, Spain; 7 Public Health Agency of Barcelona (ASPB), 08023 Barcelona, Spain; 8 CIBER de Epidemiología y Salud Pública (CIBERESP), 28029 Madrid, Spain; 9 Institut d’Investigació Biomèdica Sant Pau (IIB St. Pau), 08025 Barcelona, Spain; 10 Istituto di Ricerche Farmacologiche Mario Negri IRCCS (IRFMN), 20156 Milano, Italy; 11 Institute for Social Marketing and Health, Faculty of Health Sciences and Sport, University of Stirling (UNISTIR), Stirling FK9 4LA, UK; 12 Institute of Public Health, The American College of Greece, GR-153 42 Athens, Greece; 13 Fondazione IRCCS Istituto Nazionale dei Tumori (INT), 20133 Milan, Italy; 14 Istituto per lo Studio, la Prevenzione, e la Rete Oncologica (ISPRO), 50139 Firenze, Italy; 15 Department of Economics, Polytechnic University of Cartagena (UPCT), 30202 Cartagena, Spain; 16 School of Informatics, University of Edinburgh, Edinburgh EH8 9YL, UK; 17 European Network on Smoking and Tobacco Prevention (ENSP), 1050 Brussels, Belgium

**Keywords:** smokefree laws, COPD, asthma, particle exposure, sensors, outside areas

## Abstract

Smokefree laws are intended to protect against second-hand smoke (SHS) in outdoor areas. We examined if exposure to PM2.5 particles in outdoor smoking areas changed breathing rates in 60 patients with asthma (*n* = 30) or with COPD (*n* = 30), in an open, non-randomised, interventional study model in Czechia, Ireland and Spain. The patients wore a PM2.5 particle monitor (AirSpeck) and a breath monitor (RESpeck) for 24 h to determine changes in breathing rates (Br) at rest and during a visit to an outside smoking area. Spirometry and breath CO were measured before and the day after visiting an outdoor smoking area. The PM2.5 levels at the 60 venues were highly variable, ranging from ≥2000 µg/m^3^ (in 4 premises) to ≤10 µg/m^3^ (in 3 premises, which had only a single wall in the structure). At 39 venues, the mean PM 2.5 levels were ≥25 µg/m^3^. The breathing rate changed significantly in 57 of the 60 patients, resulting in an increase in some patients and a decrease in others. Comprehensive smokefree laws were ineffective in protecting asthma and COPD patients from exposure to high levels of SHS in outside areas of pubs and terraces, which should be avoided by these patients. These findings also support the extension of smokefree laws to outside areas.

## 1. Introduction

Smokefree legislation and policies have increased globally [1,2,3,4]. In most EU countries, smokefree laws have been implemented in public buildings and in private businesses [3,5,6]. Their aims, in general, have been to protect workers and customers from exposure to second-hand smoke (SHS) and improve health.

They have been successful in reducing exposure to SHS, with short- and long-term benefits, improving health, reducing illness, increasing smoking cessation, and denormalising smoking [4,7,8,9,10,11,12,13,14]. Specific health benefits have been shown to accrue to vulnerable populations, including children and those with underlying diseases [12,15].

Despite decreasing smoking prevalence in the last thirty years, the increased population growth in the same period has led to a significant increase in the total number of smokers. In 2019, more than 1 billion people smoked tobacco regularly, with almost 8 million deaths attributable to smoking [16].

While smokefree legislation and policies have led to a decline in smoking prevalence, as the global population grows and with an estimated 77% of the world’s population still vulnerable to SHS [17], more non-smokers are exposed to SHS hazards [16,18].

A systematic exploration of the global burden of disease attributable to SHS across 204 countries and territories from 1990 to 2019 found that SHS exposure increased the risk of tracheal, bronchus and lung cancers, breast cancer, ischemic heart disease, chronic obstructive pulmonary disease (COPD), stroke, lower respiratory infections and diabetes mellitus [18]. That analysis [18] also found that the number of years lived with disabilities (YLDs) as a result of SHS more than doubled between 1990 and 2019.

Regarding outdoor areas, the subject of this study, the details of the smokefree laws vary, and result in variable exposures. At entertainment venues, such as pubs, bars and clubs, an allowance is usually made within laws to permit an area (or terraces) outside the main premises where smoking is allowed, provided that these areas are separate and are not complete buildings to allow for increased ventilation, and that that there is no commercial activity [19].

However, it has become obvious in many instances that these smoking areas allow for the accumulation of SHS and cannot be considered safe [20,21]. Since we now also accept that there is no safe level of SHS exposure [22], it can be expected that the exposure in those areas causes adverse health effects in the long term. Nasal and oral sensory symptoms have been observed, and lung function measurements have shown deterioration from long-term exposure to SHS [23,24,25,26,27].

Chronic respiratory diseases cause an important worldwide health burden. It was estimated that, in 2017, they were the third leading cause of death, behind cardiovascular diseases and neoplasms. Globally, there were 3,914,196 deaths due to chronic respiratory diseases in 2017, an increase of 18.0% since 1990 [28].

While scientific evidence has accumulated linking SHS exposure to longer-term adverse health outcomes, including respiratory outcomes in children and adults, acute cardiovascular effects and lung cancer [13,27,29,30,31,32,33], knowledge about acute health effects of SHS on respiratory disease patients is scarce, although the present knowledge suggests that acute adverse SHS effects are the most likely to be seen in the upper or lower respiratory system or the cardiovascular system [27,31,34]. Furthermore, subjects with underlying diseases may be more likely to be more susceptible to acute effects, in addition to their increased risk of adverse long-term effects from SHS exposure [12].

The negative effects of SHS on respiratory function are thus well established. Moreover, it is well documented that SHS from combustible tobacco smoke outdoors results in poorer outdoor air quality [35,36]. With these known increased exposure and long-term health effects, we decided to monitor short-term exposure to SHS in outdoor areas and acute breathing responses of subjects with known doctor-diagnosed common respiratory diseases, asthma and COPD. Because of the possible, but undocumented, acute effects, only subjects who routinely visited outside smoking areas as part of their normal social life were considered for the inclusion.

Three countries with statutory comprehensive smokefree laws, which have been in place for varying lengths of time—Czechia, Ireland and Spain—were selected. Ireland introduced its comprehensive smokefree laws in 2004 and was the first country in the world to do so; Spain introduced its smokefree laws initially in 2008 and strengthened them in 2012; and Czechia introduced its comprehensive laws in 2016 [37,38,39]. These countries reflect a geographic and temporal spread in the EU. Their laws also allow for smoking in special areas in a variety of structures which are outside the main premises.

## 2. Methods

**Figure 1:**
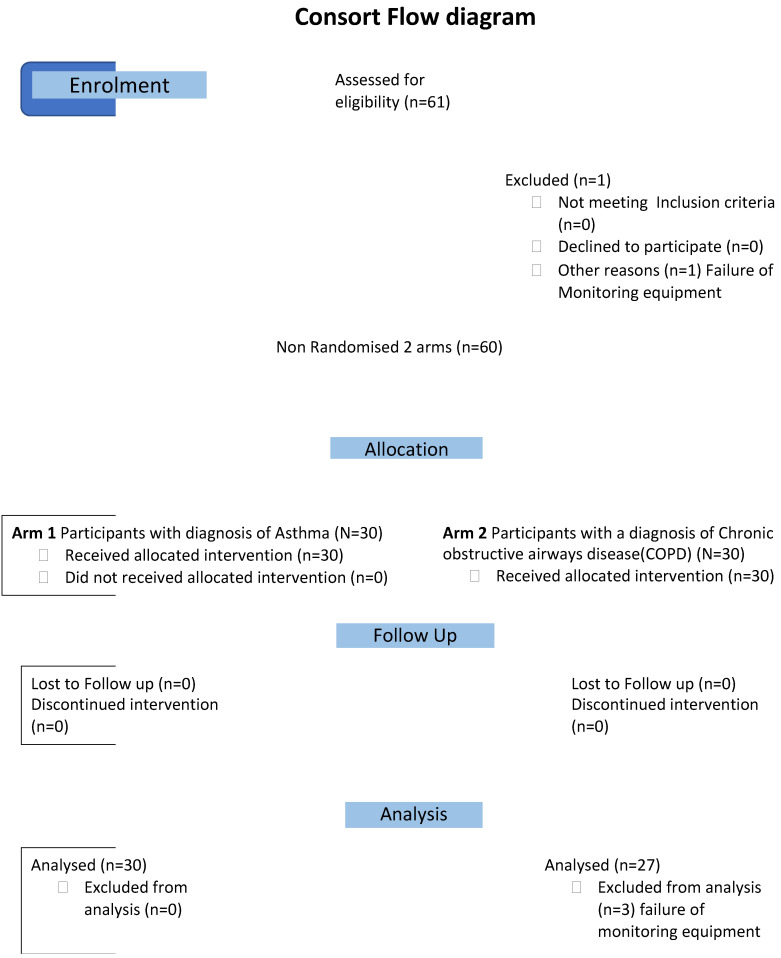
Consort flow diagram.

The study is an open, multi-centre, non-randomised, interventional study model of the acute effects of exposure to SHS in outside smoking areas in 3 EU countries with comprehensive smokefree laws. All 60 patients (Figure 1, consort flow diagram) were assessed in a similar manner, with personal monitoring of particle exposure to PM 2.5 and breathing pattern on a visit, of at least one-hour duration, to an outside area/terrace of a pub. All the measurements reported were acquired with the subjects resting for at least 15 min before visiting the venue and during exposure to SHS in a legal outside smoking area.

### 2.1. Ethics

Ethical approval was awarded in Ireland by the Dublin Institute of Technology, Research Ethics Committee (approval ref. 13.103); in Spain by Comité de Ética de La Investigación con Medicamentos del Hospital Universitario de la Princesa, Madrid (Nº de registro: 3221); and in Czechia by the Ethics Committee of the Regional Hospital in Liberic (ref no. EK/22/2018) (Supplementary file S1 uploaded).

The study protocol (also included in the Supplementary file S1 uploaded) was registered on the ClinicalTrials.gov (Accessed on 26 May 2023), with identifier NCT03074734.

### 2.2. Recruitment

Preliminary discussions were held with patient representative groups in Ireland and, following these discussions, it was decided that recruitment through contact with established chest clinics would be more appropriate than a direct approach to patients for safety, ethical and consent considerations.

The study was discussed in each of the three countries, Czechia, Spain and Ireland, with hospital staff, and copies of the full protocol were made available, as well as patient information leaflets and copies of the consent forms.

Consent: informed written consent was obtained from each subject during an interview at a specially arranged visit to the centre, where the study was explained and each patient given written information. The voluntary nature of their consent was stressed and their right to withdraw at any stage was explained.

Criteria for eligibility: fully ambulant; minimum age 18 years; sex, all; doctor-diagnosed COPD patients who were current or ex-smokers, or doctor-diagnosed asthma patients, irrespective of smoking history; and established (at the interview) that it was usual practice for each participating patient to visit outside smoking areas of pubs and bars in their usual social life.

Exclusion criteria: under 18 years of age, on oxygen therapy, pregnant, and currently undergoing treatment for an acute exacerbation of their primary condition.

### 2.3. Group Assignment

It was explained that this study followed an interventional model with single-group assignment and that there was no randomisation.

### 2.4. Details of the Intervention

Monitoring devices: AirSpeck monitors employ a light-scattering nephelometer for recording real-time PM2.5 concentration data at 10 s intervals [40]. RESpeck monitors are light-weight—17 g (incl. battery)—unobtrusive devices, which use an encapsulated tri-axial accelerometer to identify the personal mode of the subject when wearing the device, i.e., stationary, lying or mobile, which is then used to derive a reliable measure of activity, of respiratory rate and geolocation [41]. Each pair of sensor readings was communicated wirelessly using Bluetooth connectivity to a smartphone, where it was GPS-stamped for later onward transmission to a secure server dashboard for display and later offline analysis. All the exposure measurements for each of the 5 AirSpeck and 5 RESpeck monitors used were adjusted according to the calibration factor derived in experimental studies in the Edinburgh laboratory and the National Physical Laboratory, Postcode: TW110LW. The data were analysed in consultation with Edinburgh University colleagues.

National research partners in Spain and Czechia were trained by the Irish research team in the use of AirSpeck and RESpeck monitors. The study was carried out sequentially at the three centres, one in each country, over a one-year period, allowing for the same calibrated sensors to be used at each centre.

### 2.5. Patients, Protocol and Training

The study population consisted of 60 patients (30 asthma and 30 COPD patients) in Czechia (30 patients), Ireland (10 patients) and Spain (20 patients). Each patient visited their local national study centre on two occasions. During the first visit, the study was explained to each participant, both in written (information sheet) and oral communication. They completed a recruitment questionnaire to ascertain personal smoking status, other sources of exposure, average weekly attendance and SHS exposure in hospitality premises and the experience of respiratory symptoms. All consented patients were trained in the use of monitoring equipment.

Diary cards were demonstrated and explained to the patients, and they were asked to fill in details at the first visit: medication consumed, any symptoms (e.g., cough, wheeze), doctor or hospital visits, exposure to SHS and the number of cigarettes smoked (if any).

Diary card entries were also made on the day of the exposure and included a description of the premises visited, number of smokers present during exposure time, as well as any change in their use of medication required during the 24 h period or unscheduled visits to the hospital or doctor.

The participants were also asked to note the time and date when the exposure to SHS occurred in outside areas.

### 2.6. Venues

At least one visit to an outdoor smoking area was scheduled during a one-hour visit to a premise. An outdoor smoking area was defined as a place or premise, or part of a place or premise that is fully uncovered by any roof, fixed or mobile, or an outdoor place or premise that is covered by a roof, so long as not more than 50% of the perimeter (outside) is covered by a wall, windows, gate or similar.

The study subjects were asked to spend at least 15 min in the outdoor smoking area, a preferable time of 30–60 min, and 15 min at rest was desirable.

### 2.7. Measurements

The patients wore the personal monitors for 24 h to continuously measure exposure to particulate matter PM2.5, with continuous geolocalisation monitoring (AirSpeck) and a RESpeck monitor to measure the breathing rate (Br), to detect activity and any acute changes in breathing before and during exposure to SHS. To have a standardised period for the measurement of breathing rates, we selected a period of 7 min when the patient was at rest before the exposure to SHS, as defined by the RESpeck measurements, and the PM2.5 was less than 10 µg/m^3^, and compared it to breathing rates for 7 min at rest, during the exposure and when the PM 2.5 was greater than 10 µg/m^3^**.**

At the second study centre visit on the day post-exposure, all data recorded by the devices were downloaded and checked, and any diary card anomalies were addressed and clarified with the patient.

Routine pulmonary function tests consisting of forced expiratory volume in the first second (FEV_1_), forced vital capacity (FVC) and peak expiratory flow rate (PEFR) were measured at the study centre pre- and post-exposure to SHS within 24 h (and are reported elsewhere) [34].

### 2.8. Statistical Analysis

Baseline characteristics of the participating patients by their diagnosis were compared using descriptive statistics (mean, standard deviation (SD), median, interquartile range (IQR) and percentages as appropriate). The Student t-test for continuous variables and Chi-square test for categorical variables were used to determine whether there was a difference in the breathing rates among the variables of interest, and a two-tailed *p*-value, with a less than 0.05 significance threshold, was chosen for all tests. Stata v16 (Stata Corp LP, College Station, TX, USA) was used for the statistical analysis.

## 3. Results

Table 1 shows the demographic characteristics of the 60 patients. The COPD participants were older (age 63.3 ± 10.2 yrs.) than the asthmatics (46.9 ± 18.7 yrs.), and there were more women (*n* = 35) than men (*n* = 25). Of the COPD group, 21 patients (70.0%) were current smokers, as were 8 of the asthmatics (26.7%), while 15 of the 60 (25.0%) were ex-smokers. Sixteen of the asthmatics (53.3%) had never smoked. No patient reported significant changes either of maintenance medication or unscheduled visits to hospital or doctor. The number of smokers in the outdoor areas was usually fewer than five. Mainly, there were three or four walls in the smoking areas, with fewer than 20% having one or two walls. The PM 2.5 levels (Table 2) varied wildly within the smoking areas, mainly depending on the number of walls in the facility and less on the number of smokers.

### 3.1. Exposure Levels

Table 2 shows the mean and median PM 2.5 (µg/m^3^) exposure for all subjects during their visits to an outdoor smoking area (SHS exposure) and for the rest of the 24 h period (not in SHS area). While the level of exposure was greater in the SHS areas, many patients also had high exposure during the whole observed periods. It is of note that 29 of the patients were smokers.

Individual venue measurements of PM2.5 were highly variable. One area reached 2500 µg/m^3^ for a short period during venue exposure and was sustained for 15 min at ≥2000 µg/m^3^. The PM2.5 levels in 4 premises were ≥500 (1933–539) µg/m^3^, in 11 premises ≥ 200 (480–203) µg/m^3^, in 9 premises ≥ 100 (170–108) µg/m^3^, in 10 premises ≥ 40 (80.5–40.1) µg/m^3^, in 9 premises ≥ 25(39.2–25.6) µg/m^3^, in 10 premises ≥ 11 (23.28–11.0) and ≤10 (8.6–5.8) µg/m^3^ in 3 premises, which had only a single wall.

### 3.2. Respiratory Responses

The mean breathing rate (Br) (Table 3(a)) tended to be lower during exposure to SHS, ranging from 17.88 to 28.58 in the non-SHS areas at rest, and 16.46 to 27.56 during the SHS areas at rest, but the difference was not statistically significant. The pattern was similar looking at the means and medians, asthma and COPD, men and women, smokers and non-smokers (not shown in table). Exposure to SHS changed the patients’ Br. For some subjects there was a significant increase in Br during exposure to SHS and for others a significant decrease (Table 3(b)).

Table 4(a) shows the results for the overall population of Br at rest before and during SHS exposure (i.e., not differentiated by whether patients had increased or decreased Br as a result of SHS exposure), according to gender, asthma/COPD, smoking status and duration of exposure. Table 4(b,c) [42] separately examines the increased and decreased Br subgroups, for both asthmatic and COPD patients. Overall, 19 female and 9 male patients had an increased Br, and 14 female and 15 male patients had a decreased Br. In Table 4(b), we see that the Br increased significantly during SHS exposure among 17 asthmatic patients, but the increase did not reach the threshold for statistical significance in males. Among 11 COPD patients, SHS exposure significantly increased the Br for both male and female patients. In Table 4(c), we see that for those whose Br decreased during exposure, there were statistically significant changes for both male and female patients in both asthma and COPD disease subgroups.

Table 5 further examines the differences between the subgroups where the Br either increased or decreased. Table 5(a) shows differences in those where the Br either increased or decreased according to population characteristics. A younger age, female gender, lighter body weight, non-smokers, asthma, higher CAT/ACT grade and a shorter duration of exposure were more commonly associated with an increase in Br, whereas older age, male gender, heavier body weight, COPD, lower CAT/ACT grade and a longer time exposure to SHS tended to be associated with a decrease in Br, but these changes were not statistically significant.

We also examined the differences in routine pulmonary function tests between those with a decreased and an increased Br. Routine pulmonary function tests consisting of forced expiratory volume in 1 s (FEV1), forced vital capacity (FVC) and peak expiratory flow rate (PEFR) were measured at the study centre visit on the day of the pub visit, and repeated at a second study centre visit on the day following the pub visit. There were minor changes measured in lung function, which only showed a statistically significant deterioration in female asthma patients, which are reported elsewhere (34). In this study, only female asthmatics who had a decrease in the FEV1, FVC or PEFR had a statistically significant increase in the Br as a group, and this is shown in Table 5(b).

## 4. Discussion

This study confirms that exposure to SHS under the present legislation in legal outside areas in three EU countries with comprehensive smokefree laws still results in exposure to very high SHS levels [9,21]. There is no safe level of SHS [43] and chronic exposure to the SHS levels seen in this study has been shown to result in cancer, heart attacks and COPD in those who are chronically exposed [18,44]. Removal from SHS exposure in the short-to-medium term has resulted in improvements, not only in symptoms, but also in improved pulmonary function, even in asymptomatic bar workers whose pulmonary function was within normal limits [7,12,45,46]. Nevertheless, such reports of effects of short-term exposure to SHS on acute pulmonary function are scarce. We argue that any such effects are most likely to be of increased clinical importance to patients with already compromised airflow limitations. In that regard, we opted to measure the effects on breathing in patients with doctor-diagnosed asthma and COPD. We accepted only volunteer patients who had normally attended such venues where exposure to SHS was usual and had not noticed significant ill-effects on many such previous visits to pubs or bars with outdoor areas where customers are allowed to smoke. Many of the COPD patients were still smokers or ex-smokers. Of interest was also that when we approached asthma/COPD patient organisations to discuss participation, most of the members with severe diseases told us that they had abandoned visits to pubs because of SHS exposure and they did not take part in the study.

The changes in breathing rates that we recorded were complex. Nearly half of the patients increased their breathing rates, and an almost equal number decreased their breathing rate at rest in comparison with resting rates during non-exposure, and these changes were statistically significantly different. Responses in younger, lower weight, non-smoking and female patients with asthma were associated with increases in the breathing rate, while older, heavier, smoker and ex-smokers, and male patients with COPD were more likely to decrease the breathing rate. This suggests that there are disease, gender, age, weight, and smoking effects in the responses, but these were directional changes only, which did not reach statistical significance except for female asthmatics who increased rates in line with a reduction in spirometry [34]. This increased response in asthmatics is in line with the increased bronchial responsiveness of asthmatics [47], but it did not happen in all asthmatics and was not significant in males. It is known that the Br is higher in women than in men [48]. It has also been reported [47] that the change in breathing rates leads to the possibility of hypoventilation and hyperventilation, since the low and high breathing rates seen in that study are known to be associated with hypercapnia and hypoxaemia, respectively. However, we have been unable to find any previous studies testing the effect of SHS on breathing rates in patients with asthma or COPD, or in subjects without disease.

Our findings also raise the question of possible alternative mechanisms at work [49,50]. The most obvious perhaps is the different regulation of breathing apart from bronchial responsiveness. We know of the blunting of the chemical drive to breathing in chronic hypoxia regarding the response to carbon dioxide (CO_2_) [51], but we know much less about the effect of the various chemicals in SHS on the regulation of breathing in different disease states. The chemical content, concentration and dispersion of SHS are likely to be very different in different settings, in different countries [52]. The dose inhaled is likely to vary widely and, if the susceptibility also varies, then this may account for or contribute to the variance in response that we saw in this study. The study was not designed to answer this question and the variation in the patient characteristics and sample size are also unsuitable to shed light on this aspect of the results. However, the main aim of the study was to determine if SHS exposure in legal outside smoking areas was associated with measurable changes in breathing. We believe this is an important question as the rationale for smokefree bars was to protect staff and patrons from harmful exposure to SHS. This has been largely achieved inside pubs in countries with comprehensive bans on smoking, but most legislation envisions an area outside the pub supplied by the owners of the pubs where smoking is allowed. It was anticipated when framing the smokefree legislation that these areas would be such that there was negligible or no exposure to SHS of staff or non-smokers, as they would not visit these areas for any length of time, as commercial activity would not be allowed and smokers would only use them short-term. The reality is different as commercial activity has crept back into these spaces and they are visited by smokers and non-smokers. Now that we know there is no safe level of long-term exposure to SHS, it is especially important to know there are significant respiratory changes due to short-term exposure. This is particularly important for patients such as those who took part in this study, who already have an impaired pulmonary function due to disease.

## 5. Conclusions

This study in patients with asthma and COPD shows high levels of SHS exposure in outside areas of hospitality premises in the three selected EU countries with comprehensive smokefree laws. This SHS exposure was shown to have acute effects on breathing in these patients. These real-world observations of high SHS exposure and acute breathing changes suggest that all such patients be advised to avoid these areas. These findings and the known long-term adverse effects of SHS should increase the demands for an extension of smokefree laws to outside areas, abandoning designated areas and redefining a smokefree pub as an establishment where smoking is not allowed in any part of its premises.

## Figures and Tables

**Table 1:** Demographic and clinical characteristics of 60 patients, number smoking in the facility and walls in the structure (mean ± SD/*n* (%)).

	Asthma	COPD	All	
	*n* = 30	*n* = 30	*n* = 60	*p*-Value
Age in years	**46.9 ± 18.7**	**63.3 ± 10.2**	**55.2 ± 17.1**	**>0.01**
Weight in kg	75.6 ± 18.1	80.3 ± 16.5	78 ± 17.3	0.33
Sex				
Male	11 (36.7%)	14 (46.7%)	25 (41.7%)	0.30
Female	19 (63.3%)	16 (53.3%)	35 (58.3%)	
Smoking status				
Current smoker	**8 (26.7%)**	**21 (70%)**	**29 (48.3%)**	**>0.01**
Ex-smoker	**6 (20%)**	**9 (30%)**	**15 (25%)**	
Never smoked	**16 (53.3%)**	**0 (0%)**	**16 (26.7%)**	
Lives with a smoker				
Yes	7 (23.3%)	10 (33.3%)	17 (28.3%)	
No	23 (76.7%)	20 (66.7%)	43 (71.7%)	0.39
CAT/ACT score	**21.8 ± 3.6**	**12.0 ± 7.4**	**16.88**	**>0.01**
Number smoking in the outdoor smoking areas during visit				
1–5 smokers	22 (73.3%)	22 (73.3%)	44 (73.3%)	
More than 5 smokers	8 (26.7%)	8 (26.7%)	16 (26.7%)	0.77
Number of walls in the outdoor smoking area				
1 and 2	5 (16.7%)	6 (20%)	11 (18.3%)	
3 and 4	25 (83.3%)	24 (80%)	49 (81.7%)	0.44

Bold numbers indicate statistical significance at <0.05. ACT: asthma control test, range 5 to 25; CAT: COPD assessment test, range 0 to 40. Table 1 is based on data published previously [34].

**Table 2:** Exposure levels of PM2.5 µg/m^3^ during AirSpeck monitoring of 60 patients before outdoor smoking area visit (not in SHS area), and 57 patients during the outdoor smoking area visit (in SHS area).

	*n*	µg/m^3^	SD/IQR	Range (µg/m^3^)
Mean PM 2.5				
not in SHS area,	60	101.45	164.5	5.84–987.99
in SHS area,	57	233.59	359.81	5.81–1933.71
Median PM 2.5				
Not in SHS area,	60	68.40	42.2	4.51–812.59
In SHS area,	57	214.26	362.61	5.84–1913.3

**Table 3:** Mean and median breathing rates (Br) for patients at rest before (PM 2.5 < 10 µg/m^3^) and during (PM 2.5 ≥ 10 µg/m^3^) SHS exposure.

(a) Overall Population					
	*n*	Mean	SD/IQR	Range Br	
Mean breathing rates					
Not in SHS area,	60	21.66	1.91	17.88–28.58	
In SHS area,	57 *	21.57	2.51	16.46–27.56	
Median breathing rates					
Not in SHS area,	60	21.64	2.33	17.71–29.47	
In SHS area,	57 *	21.58	3.17	15.98–28.12	
**(b) Population Subgroups: Br Increased or Decreased**					
Variable	Before SHS exposure mean (SD)	During SHS exposure mean (SD)	Mean difference (95% C.I)	t (df)	*p*-value
Breathing rates increased (*n* = 28)	21.47 (1.74)	22.82 (2.29)	−1.35 (−1.80, −0.91)	−6.22 (27)	0.00
Breathing rates decreased (*n* = 29)	21.95 (2.43)	20.38 (2.79)	1.57 (1.03, 2.12)	5.93 (28)	0.00

* RESpeck Br results from three patients were technically unusable.

**Table 4:** (a) Breathing rates/minute (Br) at rest, before and during exposure for the entire population (*n* = 60). (b) Increased Br during exposure (*n* = 28) and (c) decreased Br at rest during SHS exposure (*n* = 29), according to disease and gender.

(a) Breathing Rates/Minute (Br)					
Variable	Br at Rest before Exposure Mean (SD)	Br at Rest during Exposure Mean (SD)	Mean Difference (95% C.I.)	t (df)	*p*-Value
Gender					
Male (*n* = 26)	21.49 (2.04)	20.94 (2.68)	0.55 (−0.40, 1.51)	1.20 (23)	0.24
Female (*n* = 34)	21.88 (2.18)	22.05 (2.86)	−0.17 (0.76, 0.43)	−0.57 (32)	0.57
Diagnosis					
Asthma (*n* = 30)	21.00 (1.79)	20.80 (2.24)	0.21 (−0.43, 0.;84)	0.66 (29)	0.51
COPD (*n* = 27)	22.51 (2.190	22.45 (3.16)	0.06 (−0.82, 0.94)	0.14 (26)	0.89
Smoking status					
Current smoker (*n* = 28)	22.17 (2.47)	22.14 (3.03)	0.03 (−0.70, 0.77)	0.09 (27)	0.93
Ex-smoker (*n* = 16)	21.30 (1.63)	20.70 (3.25)	0.60 (−0.90, 2.10)	0.87 (12)	0.40
Non-smoker (*n* = 16)	21.27 (1.67)	21.33 (1.82)	−0.06 (−0.93, 0.82)	−0.14 (15)	0.89
Duration time of exposure					
Up to 1 h (*n* = 21)	21.60 (2.10)	21.67 (3.30)	−0.07 (−1.13, 0.98)	−0.15 (18)	0.88
1 to 2 h (*n* = 28)	21.49 (1.60)	21.26 (2.43)	0.23 (−0.60, 1.07)	0.58 (26)	0.57
>2 h (*n* = 11)	22.48 (3.09)	22.23 (2.94)	0.26 (−0.43, 0.95)	0.83 (10)	0.43
**(b) Increase in Br**					
**Variable**	**Br at rest before exposure** **Mean (SD)**	**Br during exposure** **Mean (SD)**	**Mean difference (95% C.I)**	**t (df)**	***p*-value**
Asthma (*n* = 17)	20.83 (1.28)	21.72 (1.65)	−0.90 (−1.30, −0.49)	−4.71 (16)	0.00
Male (*n* = 5)	20.45 (1.50)	21.00 (1.83)	−0.53 (−1.21, 0.14)	−2.20 (4)	0.09
Female (*n* = 12)	21.00 (1.21)	22.04 (1.53)	−1.05 (−1.58, −0.51)	−4.32 (11)	0.00
COPD (*n* = 11)	22.28 (1.94)	24.52 (2.15)	−2.05 (−2.93, −1.19)	−5.26 (10)	0.00
Male (*n*= 4)	20.84 (1.64)	23.62 (2.46)	−2.78 (−5.19, −0.38)	−3.70 (3)	0.03
Female (*n* = 7)	23.40 (1.47)	25.03 (2.00)	−1.64 (−2.62, −0.66)	−4.11 (6)	0.00
**(c) Decrease in Br**					
**Variable**	**Br at rest before exposure** **Mean (SD)**	**Br at rest During exposure** **Mean (SD)**	**Mean difference (95% C.I)**	**t (df)**	***p*-value**
Asthma (*n* = 13)	21.23 (2.34)	19.58 (2.38)	1.65 (0.74, 2.55)	3.96 (12)	0.00
Male (*n* = 6)	21.07 (2.60)	18.63 (2.03)	2.45 (2.03)	3.40 (5)	0.02
Female (*n* = 7)	21.37 (2.33)	20.40 (2.49)	0.96 (0.19, 1.74)	3.03 (6)	0.02
COPD (*n* = 16)	22.55 (2.40)	21.03 (3.00)	1.51 (0.76, 2.27)	4.28 (15)	0.00
Male (*n* = 9)	22.64 (1.80)	21.26 (2.53)	1.38 (0.28, 2.48)	2.90 (8)	0.02
Female (*n* = 7)	22.42 (3.18)	20.74 (3.70)	1.69 (0.31, 3.10)	3.00 (6)	0.02

**Table 5:** (a) Breathing rates/minute (Br), decrease or increase, by gender, age, smoking status, weight and disease diagnosis, CAT/ACT score and the duration time of exposure in 57 patients and (b) changes in breathing rates/minute (Br), decrease or increase, in 19 female asthmatics via spirometry.

(a)				
Population		Change in Br (*n* = 57)		
Characteristics (*n* = 57)		Decreased Br	Increased Br	Total/Mean Difference (95% CI) *n*= 57
		*n* = 29 (50.88)	*n* = 28 (49.12)	
Gender			Gender	
	Male	15 (51.72)		Male
	Female	14 (48.28)		Female
Mean Age (years)		56.00 ± 17.05	Mean Age (years)	
	Male	58.27 ± 17.47		Male
	Female	54.64 ± 17.05		Female
Smoking Status			Smoking Status	
	Current Smoker	16 (55.17)		Current Smoker
	Ex-Smoker	7 (24.14)		Ex-Smoker
	Non-Smoker	6 (20.69)		Non-Smoker
Mean Weight (kg)		77.68 ± 16.00	Mean Weight (kg)	
	Male	86.18 ± 13.76		Male
	Female	68.57 ± 13.20		Female
Diagnosis			Diagnosis	
	COPD	16 (55.17)		COPD
	Asthma	13 (44.83)		Asthma
CAT/ACT Score			CAT/ACT Score	
	COPD (CAT)	10.12 ± 4.72		COPD (CAT)
	Asthma (ACT)	21.38 ± 3.42		Asthma (ACT)
Duration Time of Exposure			Duration Time of Exposure	
	Up to 1 h	7 (24.14)		Up to 1 h
	1 to 2 h	14 (48.28)		1 to 2 h
	>2 h	8 (27.59)		> 2 h
**(b)**				
**Changes in female asthmatics according to spirometry changes**		**Breathing Rates (Br)**		**Total**
		Decreased Br	Increased Br	***n* = 19**
FEV1 *n* = 19	Decrease Increase	3 (42.86) 4 (51.14)	FEV1 *n* = 19	Decrease Increase
FVC * n = 18	Decrease Increase	2 (28.57) 5 (71.43)	FVC * n = 18	Decrease Increase
PEFR ** n = 18	Decrease Increase	3 (42.86) 4 (57.14)	PEFR ** n = 18	Decrease Increase

* One FVC reading unchanged ** One PEFR reading missing.

## Data Availability

The authors are open to data-sharing of de-identified data.

## Supplementary Materials

The following supporting information can be downloaded at: https://www.mdpi.com/article/10.3390/ijerph20115978/s1, Supplementary file S1.
