# Supplementary material for: Particle Exposure Hazards of Visiting Outdoor Smoking Areas for Patients with Asthma or COPD Even in EU Countries with Comprehensive Smokefree Laws
Source: Int J Environ Res Public Health. 2023 May 28;20(11):5978. doi: 10.3390/ijerph20115978 (PMC10252725; doi:10.3390/ijerph20115978)
Supplement: Supplementary file 1 [file ijerph-20-05978-s001.zip › ijerph-2242306-supplementary.pdf]

Tackling secondhand tobacco smoke and e-cigarette emissions: exposure assessment, novel interventions, impact on lung diseases and economic burden in diverse European populations (TackSHS)

Call: H2020-HCO-2014-2015 / H2020-HCO-2015
Topic: HCO-06-2015 Global Alliance for Chronic Diseases. Prevention and treatment of lung diseases

[redacted]

This project has received funding from the European Union's Horizon 2020 research and innovation programme under grant agreement No. 681040

Disclaimer

This document was developed by the TackSHS Project Consortium and does not necessarily reflect the views of the European Commission. The European Commission is not responsible for any use that may be made of the information that contains in this document.

Acknowledgement

This document was developed under the Project TackSHS that received funding from the European Union's Horizon 2020 research and innovation programme under grant agreement No 681040.

More information

Public reports of the TackSHS Project and other information pertaining to the project are available through TackSHS public Web Site: http://www.tackshs.eu.

The ethics committee approvals from the following countries participating in the WP5 are enclosed below:

- Ireland
- Spain
- Czechepublic

Institiúid Teicneolaíochta Átha Cliath, Sráid Caoimhín, Baile Átha Cliath 8, Éire
Dublin Institute of Technology, Kevin Street, Dublin 8, Ireland
1 www.dit.ie/graduateresearchschool

SCOIL TAIGHDE IARCHÉIME / GRADUATE RESEARCH SCHOOL
Professor Mary McNamara

28th November 2016

Luke Clancy

Re: Ethical Clearance Ref 15-103 Dear Luke,

I am pleased to inform you that your minor amendment to your project:

'Tackling secondhand tobacco smoke and e-cigarette emissions: exposure assessment, novel interventions, impact on lung diseases and economic burden in diverse European populations. TACK SHS (Exposure to secondhand smoke in exempted areas/outside areas and acute health effects in patients with chronic lung disease)'

which you submitted to the Research Ethical Committee has been approved by Chair's action. The committee would like to wish you very best of luck with the rest of research project.

Yours sincerely

Dublin Institute of Technology
Research Ethics Committee

Institiúid Teicneolaíochta Átha Cliath, Sráid Caoimhín, Baile Átha Cliath 8, Éire
Dublin Institute of Technology, Kevin Street, Dublin 8, Ireland
1 www.dit.ie/graduateresearchschool

SCOIL TAIGHDE IARCHÉIME / GRADUATE RESEARCH SCHOOL
Professor Mary McNamara

3rd June 2016

Luke Clancy

Re: Ethical Clearance Ref 15-103

Dear Luke,

I am pleased to inform you that the following project:

'Tackling secondhand tobacco smoke and e-cigarette emissions: exposure assessment, novel interventions, impact on lung diseases and economic burden in diverse European populations. TACK SHS (Exposure to secondhand smoke in exempted areas/outside areas and acute health effects in patients with chronic lung disease)'

which you submitted to the Research Ethical Committee has been approved. The committee would like to wish you very best of luck with the rest of research project. If you have any further queries, please do not hesitate to contact Aisling Heyenga on (01) 402 7920 or at aisling.heyenga@dit.ie.

Yours sincerely

*Aisling Heyenga*________
Dublin Institute of Technology
Research Ethics Committee

Hospital Universitario de La Princesa
SaludMadrid

Comunidad de Madrid

COMITÉ DE ÉTICA DE LA INVESTIGACIÓN CON MEDICAMENTOS

Madrid, a 2 de noviembre de 2017

El Comité de Ética de La Investigación con Medicamentos del Hospital Universitario de la Princesa en su reunión del día 26-10-2017 (acta 18/17) después de evaluar la respuesta a las aclaraciones solicitadas del siguiente proyecto de investigación:

TITULO: WP 5 "Exposición al humo de segunda mano en áreas exentas / áreas externas y efectos agudos en la salud en pacientes con enfermedad respiratoria crónica." Versión E 2017.
TIPO: Proyecto de investigación. Convocatoria: H2020-HCO-2014-2015 / H2020-HCO-2015.
CODIGO: Estudio TACK SHS WP5 TFRI
Nº de Registro: 3221
Investigador Principal: Joan B. Soriano (Servicio de Neumología)
Decisión tomada: Aprobación (26-10-17, acta 18/17)

Este Comité de Ética de La Investigación con Medicamentos considera que tanto el proyecto de investigación como la Hoja de información al paciente y consentimiento informado son ética y metodológicamente aceptables. Asimismo, considera que los investigadores son competentes para llevar a cabo este proyecto que está enmarcado dentro de las líneas de investigación prioritarias del Hospital Universitario de La Princesa.

Hospital Universitario de la Princesa
COMITÉ ÉTICO DE INVESTIGACIÓN CLÍNICA
Madrid

Fdo.: Dra. Mª del Mar Ortega Gómez
SECRETARIA DEL CEIm

ETICKA KOMISE PRO MULTICENTRICKA HODNOCENi
Krajska nemocnice Liberec, a.s.
Ethics Commiuee of the Regional Hospital in liberec
Husova 357/ I0, 460 01 liberec I- Stare Mesto, tel/fax: 485312835, e-mail:eticka.komise nemlib.cz

STANOVJSKO ETJCKE KOMISE/Opinio11of the Ethics Committee

Nazev projektu/Fu/1 *Title of Proiect:*
Expozice pasivnimu koureni ve vybranych venkovnich prostorach, kde se kouri a akutni zdravotni dopad na pacienty s chronickym plicnim onemocnenim. /
Exposure to secondhand smoke in exempted areas/outside areas and acute health effects in patients with chronic lung disease.

Zadatel/Applicant:
MlJDr. Milada Sipkova, Plicnf ambulance, PuImo s.r.o., Klasternf 2/117, 460 01 Liberec;
tel.: +420 485 312 335; mail: pulmo.liberec@seznam.cz

Datum podani *zadosti/Date of submissio11o[tlte Applicatio11Form:*
5.2.2018

Datum jednani *EK/Date a11d time o[Etltics Committee session:*
21.2.2018

Cislo *jednaci/Refere11ce number:*
EK/22/2018

Hodnocene dokumcnty/List *of all Submitted documents:*
•Mail dated 5 February 2018
•SHS Info Sheet and consent form.docx
•Draft Data Data collection Work sheet.xlsx
•TFRI Tack SHS Appendix-2 Info Sheet and consent form.docx
•Diary+Card+Word+doc.docx
•form WP 5 data.doc
•TACK protocol DS.1.pdf

Stanovisko eticke *komise/Etltics Committee's opinion:*

Eticka komise vydava souhlasne stanovisko/ *Ethics Committee issues favourable opinion*

Datum/*Date:* 21. 2. 2018

Podpis predsedy *EK/Signature of Chairperson EC*

Krajskon mocr ce L1berec, o.s.
Husova 357/ " I Stare Mesta
•160 1 L , erec
Multicentrickci etickci komise

Rozdl!lovnik/ *list o(Distribution:*
MUDr. Milada Sipkova, Plicni ambulance

Seznam clenii eticke komise/ *List of the Ethics Committee Members:*

Jmeno a prijmeni *First name and surname*	MuV Zena *Male/ Female*	Odbomost *Specialism*	Zamestnanec zfizovatele EK' Ano Yes	Ne No	Funkce v EK *Role in EC*	Pritomen *Attendance* Ano *Yes*	Ne *No*	Hlasoval *Voted* Ano *Yes*	Ne *No*
MUDr. Pavel Koci	M/M	nefrolog/ nefrologist	[81	☐	pi'edseda/ chairman	[81	☐	[81	☐
MUDr. Michael Sip	M/M	pediatr/ pediatrician	[81	☐	tajemnfk/ secretary	[81	☐	[81	☐
MUDr. Hana Vechtova	ZIF	intenzivista/ intenzivist	[81	☐	clen/ member	[81	☐	[81	☐
MUDr. Kveta Sourkova	ZIF	intemista/ internist	[81	☐	clen/ member	[81	☐	[81	☐
MUDr. Jaroslav Sram	M/M	chirurg/ surgeon	[81	☐	clen/ member	☐	[81	☐	[81
MUDr. Marie Rissova	ZIF	onkolog/ oncologist	[81	☐	mfstopi'edseda/ vice-chairman	[81	☐	[81	☐
Mgr. Margit Kotkova	ZIF	pedagog/ pedagogue	☐	[81	nezavisly clen/ independent	[81	☐	[81	☐
Mgr. Jitka Cerhova	ZIF	pedagog/ pedagogue	☐	[81	nezavisly clen/ independent	[81	☐	[81	☐
Zdena Brabcova	ZIF	sekretafka/ secretary	[81	☐	nezavisly clen/ independent	[81	☐	rgJ	☐
MUDr. Hana Vejvarova	ZIF	pediatr/ pediatrician	☐		clen/ member	☐	rgJ	☐	rgJ

(pozn: 'Zamestnanec zfizovatele EK/ *Employee of EC appointing authority)*

Eticka komise prohla uje, ze byla ustavena a pracuje podle jednaciho i'adu v souladu se spravnou klinickou praxi (GCP) a platnymi pravnimi *pi'edpisy/The Ethics Committee hereby declares that it was established and operates in accordance with its Rules of Procedure in compliance with Good Clinical Practice and valid legal regulations:*

Ano/Yes ☐*Ne/No* *Komentai'/Comments:*

Datum/Date: 21.2.2018

Podpis pfedt o stupce
Signature of Chairperson or Vice-Chairperson of the EC

1.

Muft'cer r c
